# Transformers and Generative Adversarial Networks for Liveness Detection in Multitarget Fingerprint Sensors

**DOI:** 10.3390/s21030699

**Published:** 2021-01-20

**Authors:** Soha B. Sandouka, Yakoub Bazi, Naif Alajlan

**Affiliations:** Computer Engineering Department, College of Computer and Information Sciences, King Saud University, Riyadh 11543, Saudi Arabia; 439204384@student.ksu.edu.sa (S.B.S.); najlan@ksu.edu.sa (N.A.)

**Keywords:** fingerprint, liveness detection, generative adversarial network, transformer

## Abstract

Fingerprint-based biometric systems have grown rapidly as they are used for various applications including mobile payments, international border security, and financial transactions. The widespread nature of these systems renders them vulnerable to presentation attacks. Hence, improving the generalization ability of fingerprint presentation attack detection (PAD) in cross-sensor and cross-material setting is of primary importance. In this work, we propose a solution based on a transformers and generative adversarial networks (GANs). Our aim is to reduce the distribution shift between fingerprint representations coming from multiple target sensors. In the experiments, we validate the proposed methodology on the public LivDet2015 dataset provided by the liveness detection competition. The experimental results show that the proposed architecture yields an increase in average classification accuracy from 68.52% up to 83.12% after adaptation.

## 1. Introduction

**Figure 1:**
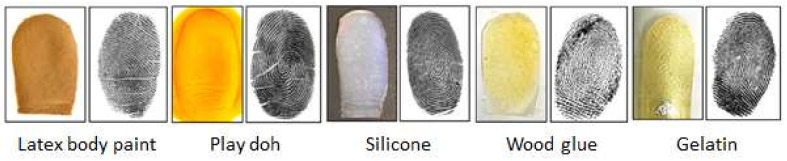
Different examples of spoof fingerprints fabricated using different materials. The photograph of the fabricated fingerprint is on the left, and the captured fingerprint image using Crossmatch sensor is on the right [4].

Biometric systems aim to recognize individuals based on physiological and behavioral characteristics such as face, iris, retina, fingerprint, hand geometry, voice, keystroke, and others. Fingerprints are one of the most used biometrics due to their advantages, such as high accuracy, collectability, uniqueness, and permanence. The fingerprint-based biometric system is widely used for various day-to-day applications such as mobile payments, international border security, and financial transactions. The rapid growth of fingerprint-based biometric systems has made them vulnerable to numerous attacks, mainly presentation attacks (PAs) [1]. Presentation attacks can be defined as the “presentation to the capture device with the goal of interfering with the operation of the biometric system” [2]. An artifact can be gummy fingers, 2D or 3D printed fingerprint targets, altered fingerprints, and cadaver fingers, an artifact also known as a spoof [3]. Usually, spoofed fingerprints are fabricated from commonly available materials such as gelatin, silicone, Play-Doh, wood glue, etc. Figure 1 shows different examples of spoof fingerprint samples created by different artificial materials, such as wood glue, gelatin, Play-Doh, and silicon [4].

Presentation attack detection (PAD) is an automated process for detecting and preventing PAs in a biometric system. It aims to discriminate between the bona fide (i.e., real or live) biometric samples and PA (i.e., artifact) samples. There are different literature methods used for fingerprint PAD and they are classified as hardware-based or software-based approaches. 

In the hardware-based approaches, an additional sensor is added to detect the physical characteristics of the fingerprint, such as temperature, blood pressure, or heartbeat [5,6,7]. Hardware-based methods can be more accurate than software-based ones, but they are more complex and expensive. It is also difficult to update the hardware devices when the attacker fabricates a new type of fake fingerprint. Thus, software-based approaches have gained increasing attention. Software-based approaches rely on image processing methods to extract features from the fingerprint images. The existing software-based approaches can be based on either handcrafted or deep learning features. 

The earliest software-based methods proposed for liveness detection are based on handcrafted features (e.g., anatomical, physiological, or texture-based features [1]). However, the related feature-extractor methods include local binary patterns (LBP), local phase quantization (LPQ) [8], binarized statistical image features (BSIF), Weber local binary descriptor (WLBD). Nikam and Agarwal [9] were among the first studies that analyze the handcrafted features. They proposed a method that combines the LBP and wavelet transformation. First, they extracted LBP histograms to capture textural features. Then, wavelet energy features are used to characterize ridge frequency and orientation information. Finally, they used hybrid classifiers for classification. Xia et al. [10] proposed a novel descriptor called Weber local binary descriptor (WLBD), which uses the local binary differential excitation component to extract intensity variance features, and uses the local binary gradient orientation component to extract orientation features. Ghiani et al. [8] proposed a set of features based on a textural analysis for detecting liveness using the local phase quantization (LPQ), which is insensitive to blurring effects. The proposed method showed promising results.

Recently, deep learning-based approaches have boosted the classification accuracies. To improve the detection process and prevent fingerprint PAs many solutions based on convolutional neural networks (CNN) proposed [4]. One of the first works based on deep learning approaches is presented by Nogueira et al. [11], where they proposed many models for fingerprint liveness detection. They firstly utilized the pretrained CNN-VGG and CNN-Alexnet models and fine-tuned them using fingerprint images. The pretrained CNN-VGG model gives state-of-the-art results with an overall accuracy of 95.51%, which made them win first place in the fingerprint liveness detection competition 2015. Chugh et al. [1] proposed a CNN-based approach for fingerprint liveness detection using the Inception-v3 model trained on local patches of fingerprint images centered around fingerprint minutiae points. This model achieved an overall accuracy of 98.61% on LivDet 2015 dataset compared to 95.51% [11] achieved by the winner of the LivDet 2015 competition.

In another two works, Nguyen et al. [12] and Park et al. [13] proposed a CNN-based model using the fire module of SqueezNet. Nguyen et al. [12] proposed a model called fPADnet, which consists of SqueezeNet Fire and Gram-K modules to extracts texture information of fingerprint images to distinguish spoof fingerprints. The proposed models are independent of input sizes and significantly reduce network size, processing time, and memory requirements.

Kim et al. [14] proposed a system to generate artificial fingerprints and detect fake fingerprints using deep neural networks. They used different architecture of a generative adversarial network (GAN) to generate the fingerprints. For presentation attack detection, they used a convolutional layer network followed by the fire module of SqueezNet. The presented algorithm for presentation attack detection gives better performance than the existing algorithms in terms of accuracy and processing time. 

Zhang et al. [5] proposed a special residual-based network for fingerprint PAD, called Slim-ResCNN, based on the utilization of improved residual blocks. At first, they used the statistical histogram to remove the blank background and center of gravity and select important local patches from the fingerprint image. Then they applied the modified fingerprint images to the proposed Slim-ResCNN approach. The proposed approach won first place in the fingerprint liveness detection competition 2017 with an overall accuracy of 95.25%. In a recent work, a new fingerprint PAD method based on the fusion of fingerprint with a more secure biometric modality (such as the face, ECG, and fingerprint dynamics) were proposed in the literature. M. Jomaa et al. [15] proposed an end-to-end deep learning-based fusion neural architecture between a fingerprint and electrocardiogram (ECG) signals. The proposed method uses EfficientNets for fusing ECG and fingerprint feature representations. 

One of the limitations of the existing fingerprint PAD algorithms is the poor generalization performance across unknown spoof materials and unknown sensors. Many researchers tried to solve accuracy degradation in fingerprint PAD in the case of unknown materials. González-Soler et al. [16] proposed a new fingerprint spoof generalization by fusing three different feature encoding of dense features, mainly Bag-of-Words (BoW), Fisher Vector (FV), and Vector Locally Aggregated Descriptors (Vlad). Then, they used a support vector machine (SVM) to classify the encoded features. The proposed approach won first place in the fingerprint liveness detection competition 2019 [17] with an overall accuracy of 96.17%. Engelsma and Jain [18] proposed a one-class classifier using three GANs based on DCGAN architecture trained on bona fide images acquired with the RaspiReader sensor for fingerprint PAD. In another work, Chugh and Jain [4] presented a universal material generator, a style-transfer-based method, to improve generalization ability. Their proposed method improved the cross-material and cross-sensor generalization performance.

Since the appearance of GANs in 2014, many computer vision tasks adopted the concept of GANs such as image-to-image translation [19,20,21], image style transfer [22,23], image generation [23,24], image super-resolution [25], scale-specific face generation [26], face completion [27], image matting [28], image painting and colorization [29,30], unsupervised learning [31], and semisupervised learning [32].

The original GANs basically consists of two competing neural network models. One is called generator *G*, and the other is called discriminator *D*. The two networks are competing with each other through a two-player minimax game. The generator is trained to fool the discriminator from differentiating between real images and generated fake images *G*(*z*). In contrast, the discriminator is optimized in order to distinguish the fake data from the real ones [33].

(1) $$\underset{G}{\min}\;\underset{D}{\max}\;V\;\left(D,G\right)=E_{x∼P_{data}\left(x\right)}\left[\log D\left(x\right)\right]+E_{x∼P_{z}\left(z\right)}\left[\log\left(1-D\left(G\left(z\right)\right)\right)\;\right]$$

The image-to-image translation goal is to convert an image in the source domain to a corresponding image in the target domain. Different image-to-image translation approaches have been proposed in the literature depending on whether aligned image pairs (paired) such as Pix2Pix [19] or two sets of (unaligned) sets such as CycleGAN [21], DiscoGAN [22], and UNIT [34] are used. CycleGAN [21] is a state-of-the-art approach for the unpaired image to image translation. It performs a bidirectional image to image translation between two sets of images despite every image having vastly different compositions.

In this work, we aim to address the poor generalization ability of fingerprint PAD over multiple sensors and materials by proposing a method based on transformers and GANs. In a first step, in order to reduce the distribution discrepancy between the source and target samples, we learn a mapping function from real/fake samples of the source sensor to few real samples coming from multiple target sensors using CycleGAN. Then, we train a hybrid network composed of a pretrained CNN coupled with a transformer on the original training samples of the source sensor in addition to their augmented version obtained from CycleGAN. In the experiments, we validate the proposed method on the LivDet 2015 dataset. 

The remaining of the paper is organized as follows. Section 2 describes the proposed method. The experimental settings and results are presented in Section 3. In Section 4, we present discussions. Finally, the conclusions are presented in Section 5.

## 2. Proposed Methodology

**Figure 2:**
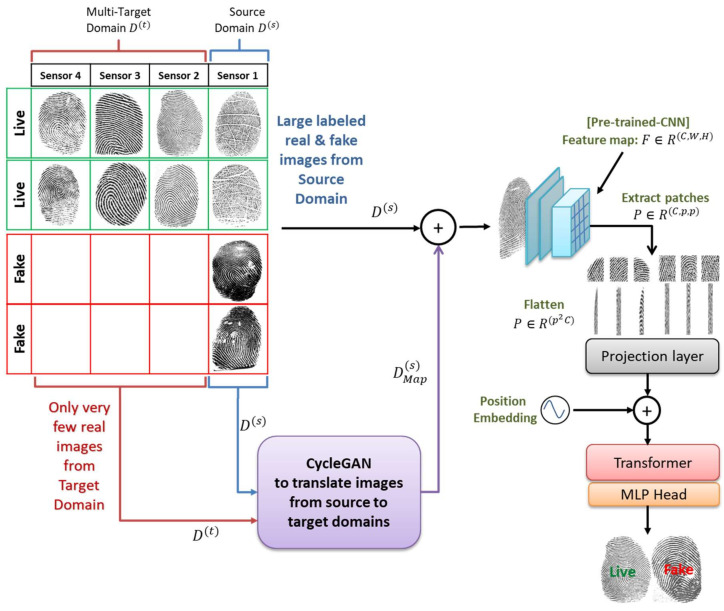
The architecture of the proposed method.

The overall architecture of the proposed method is shown in Figure 2. It contains three main components, which are described below: a CNN backbone to extract a compact feature representation of a fingerprint, a transformer, and a domain conversion with GAN to generate additional images synthetically.

### 2.1. Backbone CNN

The method uses EfficientNets as a CNN backbone [35] for generating the feature representations of a fingerprint. These models use a simple and highly effective compound coefficient to uniformly scale each network’s dimension, such as width, depth, and resolution, with a fixed ratio. EfficientNet baseline architecture is developed using a neural search architecture, which optimizes both accuracy and FLOPS. In addition, these networks use the mobile inverted bottleneck convolution (MBConv) as the basic building block. These blocks use an attention mechanism based on squeeze and excitation block to improve feature representations. Global average pooling (GAP) is applied after each block to squeeze the entire receptive field into one channel embedding. Then the excitation remaps the one channel into more channels embedding. It is worth noting that these networks use Swish activation function instead of ReLU. 

If we assume that we have an initial fingerprint image with three color channels $X_{i}\in R^{\left(C,H_{\circ },W_{\circ }\right)}$, where $\left(H_{\circ },W_{\circ }\right)$ is the resolution of the initial image and C is the number of channels. Then, feeding this image to the CNN backbone will result in a lower-resolution feature map $F\in R^{\left(C,W,H\right)}$.

### 2.2. Transformer Encoder

On the top of the resulting low-resolution feature map F we mount a transformer encoder. The latter is a sequence transduction model based entirely on attention, it was introduced in 2017 by Vaswani et al. [36]. It has become the standard model used in the field of natural language processing (NLP). The transformer replaces the recurrent layers most commonly used in encoder–decoder architectures with multiheaded self-attention. Recently the authors in [37] modified the standard transformer to be used for image classification tasks. Basically, the image is divided into a sequence of patches and used as input for the transformer. In our case, the features from the CNN backbone are passed through the transformer encoder, together with a spatial positional encoding $E_{pos\;}$ that is added to queries and keys of the multihead self-attention layer.

More specifically, we feed the low-resolution feature map $F\in R^{\left(C,W,H\right)}$ to the transformer encoder to obtain the input sequence. First, we split the feature map into fixed-size patches $P\in R^{\left(C,p,p\right)}$, where $\left(p,p\right)$ is the resolution of each patch. Then, we flatten the spatial dimensions of the feature map and project it to the dimension $P\in R^{\left(p^{2}C\right)}$. The output of this projection is called patch embeddings. Finally, the patch and position embeddings are added, and the resulting sequence of vectors is given as an input to the transformer encoder.

(2) $$z_{0\;}=\;\left[x_{p}^{1}E;x_{p}^{2}E;\ldots ;\;x_{p}^{N}E\right]+E_{pos\;}$$

**Figure 3:**
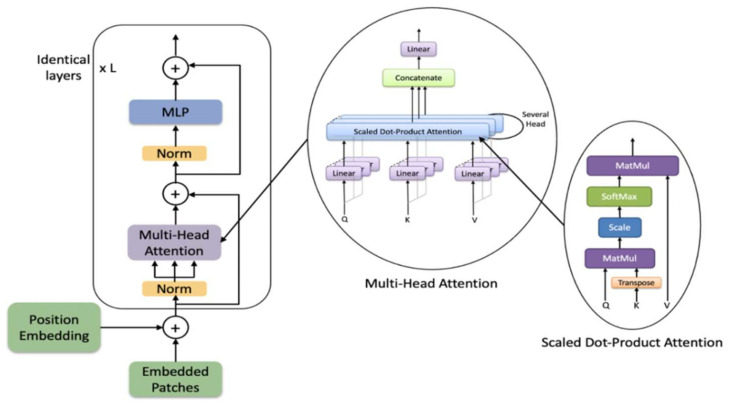
The architecture of the transformer encoder.

The transformer consists of alternating layers of multiheaded self-attention (MSA) and MLP blocks, as shown in Figure 3. Before each block of the transformer a Layernorm (LN) is applied, and residual connections after every block.

(3) $$z_{l}=\mathrm{MSA}\left(\mathrm{LN}\left(z_{l-1}\right)\right)+z_{l-1},\;\;l=0,\ldots ,L$$

where L is the number of blocks in the transformer and $z_{l}$ is the output of the lth block.

The MSA module is the concatenation of multiple layers of scaled dot-product attention operations run in parallel, called “heads”. The scaled dot-product attention function receives a query $\left(Q\right)$, key $\left(K\right)$, and value $\left(V\right)$ as an input. The dot product of all input is performed and scaled by $\left(\frac{1}{\sqrt{d_{k}}}\right)$ then a softmax function is applied to obtain the output matrix as described below:

(4) $$Attention\left(Q,K,V\right)\;=softmax\left(\frac{Q\;K^{T}}{\sqrt{d_{k}}}\right)V$$

The multihead self-attention function receives as input a multiple set of queries $\left(Q\right)$, keys $\left(K\right)$, and values $\left(V\right)$ generated based on the same input embeddings X. Then a scaled dot-product attention is performed on each set in parallel. Then, the outputs are concatenated and projected linearly to get a final output as described below:

(5) $$MultiHead\;\left(Q,K,V\right)=Concat\left(head_{1},\;\ldots ,head_{h}\right)W^{o}$$

(6) $$head_{i}=Attention\left(QW_{i}^{Q},KW_{i}^{K},VW_{i}^{V}\right)$$

where $W^{o}$ is the final projection matrix and $W_{i}^{Q}\in {\mathbb{R}}^{d_{model}xd_{q}}$, $W_{i}^{K}\in {\mathbb{R}}^{d_{model}xd_{k}}$, and $W_{i}^{V}\in {\mathbb{R}}^{d_{model}xd_{v}}$ for i=1,2, …, h are the projection matrices and they are independent in different heads.

### 2.3. Classification Layer

On top of the transformer, we use an average pooling layer to pool the transformer sequence into one feature. Then we feed this feature to a fully connected layer (*FC*) with sigmoid activation function to determine the final fingerprint class, i.e., live or fake.

(7) $$p\left(X_{i}\right)\;=\;FC\left(LN\left(z_{L}\right)\right)$$

### 2.4. Domain Conversion with GAN

As mentioned previously, we aim to improve the cross-sensor and cross-material performance. For such purpose, we employ CycleGAN network architecture to synthetically generate additional images that were transformed from the source domain to the target domain to train a finger presentation attack detector. 

**Figure 4:**
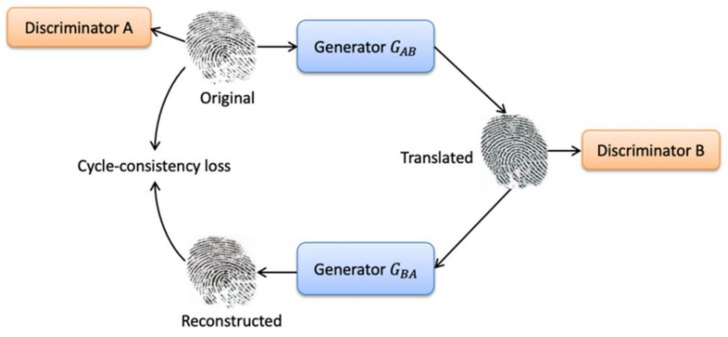
The architecture of CycleGAN.

Let us assume that we have a set of fingerprint images from source domain $D^{\left(s\right)}={\left\{X_{i},y_{i}\right\}}_{i=1}^{N}$; it consists of N samples divided into real fingerprint images and fake fingerprint images. $X_{i}$ represents the input fingerprint image, and $y_{i}$ is a binary label indicating if a fingerprint is real or fake. In this study, we used CycleGAN to generate additional images that were transformed from the source domain to the target domain. To this end, the dataset becomes $D=D^{\left(s\right)}\cup D_{Map}^{\left(s\right)}$, where $D^{\left(s\right)}$ is the fingerprint images from the source domain and $D_{Map}^{\left(s\right)}$ is the fingerprint images after adaptation. Figure 4 shows the architecture for domain translation in fingerprint images.

The architecture of CycleGAN consists of two generators $\left(G\;\mathrm{and}\;F\right)$ and two adversarial discriminators $\left(D_{X}\;\mathrm{and}\;D_{Y}\right)$. It uses cycle consistency to translate images between two different domains $\left(X\;\mathrm{and}\;Y\right)$ of unpaired images. The first generator G: X→Y: maps source images from domain $\left(X\right)$ to target images $G\left(Y\right)$ and minimizes the adversarial loss. The second generator F: Y→X: maps target images from domain $\left(Y\right)$ to the source images $F\left(X\right)$ and minimizing a second adversarial loss. The adversarial loss is:

(8) $$L_{GAN}\left(G,D_{Y},X,Y\right)\;=E_{y∼P_{data}\left(y\right)}\left[\log D_{Y}\left(y\right)\right]+E_{x∼P_{data}\left(x\right)}\left[\log(1-D_{Y}\left(G\left(x\right)\right)\right]$$

Each generator has a corresponding discriminator, which basically distinguishes between real and synthesized images. Each generator and discriminator are updated using cycle consistency loss. Cycle consistency loss compares a source image to the generated target image and calculates the difference between them. We should arrive where we started when we translate from one domain to another and back again, $F\left(G\left(X\right)\right)\approx X\;\mathrm{and}\;G\left(F\left(X\right)\right)\;\approx X\;$ [15]. The cycle consistency loss is:

(9) $$L_{cyc}\left(G,F\right)\;=\;E_{x∼P_{data}\left(x\right)}\left[∥F\left(G\left(x\right)\;\right)-x{∥}_{1}\right]+E_{y∼P_{data}\left(y\right)}\left[∥G\left(F\left(y\right)\;\right)-y{∥}_{1}\right]$$

The full objective of CycleGAN is:

(10) $$L\left(G,F,D_{X},D_{Y}\right)\;=L_{GAN}\left(G,D_{Y},X,Y\right)\;+\;L_{GAN}\left(F,D_{X},Y,X\right)\;+\;\lambda \;L_{cyc}\left(G,F\right)$$

where *λ* controls the relative importance of the two objectives. CycleGAN aims to solve:

(11) $$G^{*},F^{*}=\arg\;\underset{G,F}{\min}\;\underset{D_{X},D_{Y}}{\max}\;L\left(G,F,D_{X},D_{Y}\right)$$

Each CycleGAN generator consists of convolutional layers, residual blocks, and transpose convolutional layers. The input image is passed into three convolution layers to extract features and reduces the representation by 1/4th of the actual image size. Then, the extracted features pass through six or nine residual blocks based on the input image size. The output is then passed into two fractionally-strided convolutions to increase the size of representation to the original size. Finally, one convolution is used to map features to RGB. The discriminator uses the architecture of the PatchGAN discriminator. It consists of five convolution layers followed by InstanceNorm and LeakyReLU layer with k filters and stride 2. In the first layer, InstanceNorm does not apply. Finally, to produce a 1-dimensional output a convolution operation is applied.

In Algorithm 1, we provide the main steps for training the proposed architecture for domain adaption.
**Algorithm 1**Input: Source images $D^{\left(s\right)}={\left\{X_{i},y_{i}\right\}}_{i=1}^{N}$, Target images $D^{\left(T\right)}$ Output: fingerprint class, i.e., live or fake.Set parameters:Adam optimizer: learning rate: 0.0003Batch size: = 25Patch size p=7Layers of the transformer L=2Dimension of the fully connected layer *FC* = 128Train a CycleGAN to translate $D_{Map}^{\left(s\right)}$ from $D^{\left(s\right)}$ [we use the default configuration of the network [21]]Use the new dataset $D=D^{\left(s\right)}\cup D_{Map}^{\left(s\right)}$ to train the hybrid network composed of a backbone CNN and a Transformer for 50 iterations.Feed target images to the trained hybrid network to classify them as live or fake.


## 3. Experimental Results

### 3.1. Dataset Description

LivDet 2015 Dataset is a public dataset provided by the Liveness Detection Competition (LivDet 2015) [38]. This dataset includes images acquired from four different optical sensors; Green Bit, Biometrika, Digital Persona, and Crossmatch; divided into training and testing parts. There are more than 4000 images for each of these sensors with varying sizes, as shown in Table 1.

**Figure 5:**
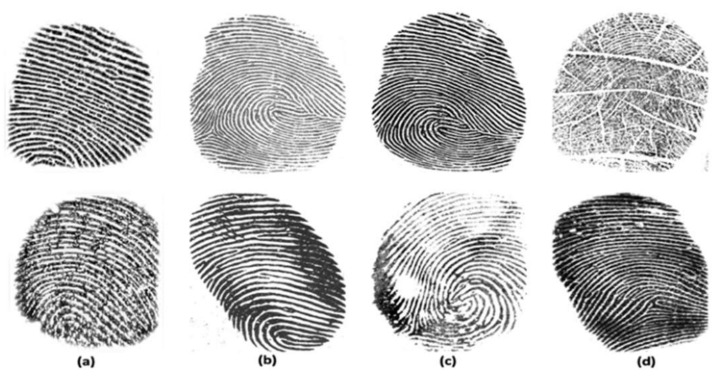
Sample images of LivDet2015 dataset. Live samples are in the top row and fake samples are in the bottom row. Samples are from Digital Persona (**a**), Green Bit (**b**), Biometrika (**c**), and CrossMatch (**d**) sensors.

This dataset includes images for live fingers that were acquired in different modes in order to mimic real scenarios. Images for each finger were acquired in normal mode, with wet and dry fingers and with high and low pressure. The dataset also includes spoof images obtained using a cooperative method. Figure 5 shows sample images from the LivDet2015 dataset. The spoof material used in Green Bit, Biometrika, and Digital Persona datasets are Ecoflex, gelatin, latex, wood glue, a liquid Ecoflex, and RTV (a two-component silicone rubber) and the Crossmatch dataset uses Play-Doh, Body Double, Ecoflex, OOMOO (a silicone rubber), and a novel form of gelatin, as shown in Table 2.

### 3.2. Dataset Preprocessing

**Figure 6:**
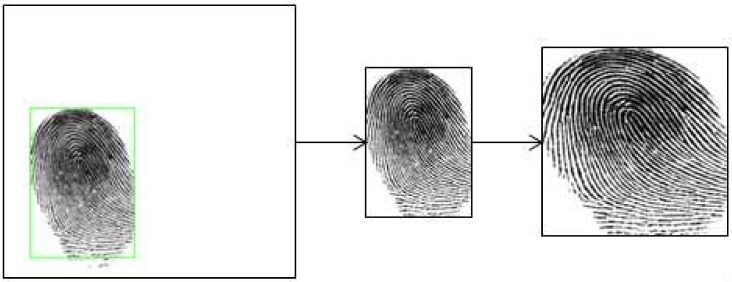
Steps of preprocessing. The original fingerprint image is from Crossmatch in LivDet 2015. Region of Interest (ROI) extraction is firstly applied to obtain the fingerprint foreground region and then resize the image.

Before we use the fingerprint images to evaluate the proposed approach, we preprocessed the images by extracting the region of interest (ROI) and then image resizing. The ROI extraction was done to avoid the effect of the white background area on efficiency and performance. We applied Gaussian blur filter to reduce the image noise level and then we used Otsu’s threshold to extract the fingerprint foreground region from the white background. Second, we applied morphological operations with a small kernel to remove noise. Then we cropped the resulting ROI images and resized them to 224 × 224 pixels as shown in Figure 6.

### 3.3. Experiment Setup and Performance Metrics

To validate the proposed methodology, we performed several experiments on the LivDet2015 dataset. All experiments were implemented in Python with the PyTorch library using a PC workstation having a Core i9 processor with a speed of 3.6 GHz, 64 GB of memory, and a GPU (with 11 GB GDDR5X memory). We used Adam optimizer as an optimization technique for training the network with the learning rate set to 0.0003 and a batch size of 25, and the number of training epochs was set to 50. We adopted the original architecture for CycleGAN, where the generator consists of three convolution layers, several residual blocks, two fractionally-strided convolutions with stride 1⁄2, and one convolution layer [21].

For performance evaluation, we used the standard measures proposed by Liveness Detection Competitions [38], with compatibility with ISO standard [39] such as:

- Accuracy: rate of correctly classified live and fake fingerprints.
- Average Classification Error (ACE):

(12) $$ACE=\;\frac{FerrLiv+FerrFake}{2}$$

where FerrLive is the rate of misclassified live fingerprints, which is equivalent to the Bona Fide Presentation Classification Error Rate (BPCER). FerrFake is the rate of misclassified fake fingerprints, which is equivalent to the Attack Presentation Classification Error Rate (APCER) [39].

#### 3.3.1. Experiment 1: Full Supervised Classification

The first experiment was performed to evaluate the effectiveness of the proposed method regarding detecting PAs and comparing our results with previous state-of-the-art methods. In this regard, we trained the CNN backbone and the transformer in a full supervised mode on LivDet 2015 dataset. Then, we tested the model using the testing part of the dataset. The network was trained using the following parameters: the learning rate was initialized to 0.0003; the transformer model’s depth was 2; the number of epochs was 50. We used different data augmentation techniques to boost the network’s robustness to the possible variations in fingerprint images. We used simple image augmentation, which consists of mainly rotations and zooming, and CutMix augmentation [40]. This last is a combination of CutOut and MixUp operation, it consists of cut and paste patches among the training images and mixing the ground truth labels in proportion to the area of patches in the images. The simple augmentation is done by zooming on a scale of 1.1 and 2 and rotating left and right by 25 degrees.

We can see from the results in Table 3 that the proposed network yields promising detection accuracies. It achieves an overall classification accuracy of 97.70% in the best scenario competing with the best accuracy of the methods from the LivDet 2015 competition [38] and previous state-of-the-art methods. We achieved better results than the proposed fingerprint network by M. Jomaa et al. [15], which is also based on EfficientNet. Moreover, the overall classification accuracy of the best scenario 97.70% is competing with 98.61% obtained by Chugh et al. [1]. Yet, we recall in this work that we are interested in the cross-sensor setting introduced in the next experiments. 

The accuracy of individual sensors shows that the Crossmatch sensor is less challenging compared to other sensors as it achieves the highest accuracy of 98.30%. On the other side, the Digital Persona sensor has difficulty learning and the sensor always achieves a relatively lower accuracy of 94.76%. Furthermore, the Green bit and Biometrika sensors achieve moderately high accuracies of 96.72% and 95.44%, respectively. 

During this experiment, we tested the effect of dataset augmentation and patch size on classification accuracy. We can see that the augmentation improves the overall classification accuracy by 1.39% using simple augmentation (rotations and zooming) and by 0.82% using the CutMix augmentation strategy. The simple augmentation performs better on the Green bit, Biometrika, and Digital Persona sensors, where the classification accuracy in the three sensors (98.44%, 97.68%, and 96.36%) is increased by 1.72%, 2.24%, and 1.6%, respectively. However, the CutMix augmentation strategy performs better on the Crossmatch sensor, where the classification accuracy increased by 0.71%. Moreover, we tested the proposed network when the patch size was 4, and with simple augmentation, this did not show good improvement in the classification accuracy. In the next experiments, we used the proposed network with simple augmentation since it achieved the best overall classification accuracy of 97.70%.

#### 3.3.2. Experiment 2: Generalization Ability

In the second experiment, we trained the CNN backbone and vision transformer in cross-sensor settings. Then we tested the resulting models on images from each sensor individually. We trained the network for sufficient 50 iterations. The reported results in Table 4 show the degradation in the accuracy in the case of the cross-sensor. We can see that when we test the network using the same sensor used for training, we get high classification accuracy even though we have unknown materials. However, when we trained the network on images from one sensor and tested on images from another, cross-sensor setting, we get low classification accuracy. For example, when we trained the proposed network on images from the Green Bit sensor and tested on Green Bit sensor images, we get a high classification accuracy of 97.56%. On the other hand, the accuracy drops significantly when we test the network using images from other sensors 83.68%, 66.60%, and 63.97% for Biometrika, Digital Persona, and Crossmatch, respectively.

**Figure 7:**
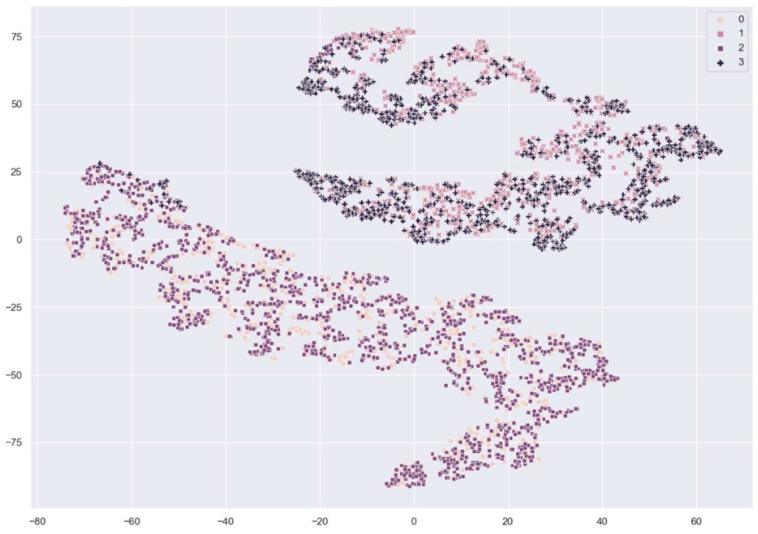
The distribution of fingerprint samples before adaptation within the same sensor. 0: train live, 1: train fake, 2: test live, 3: test fake samples from Crossmatch sensor.

**Figure 8:**
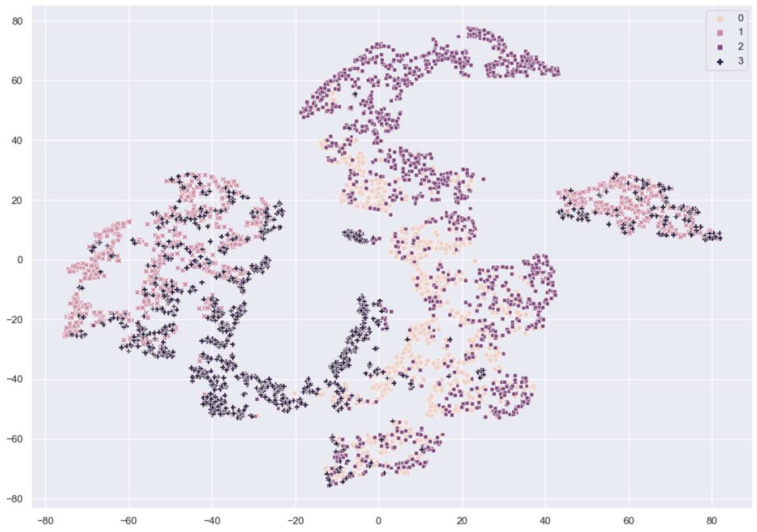
The distribution of fingerprint samples before adaptation in the cross-sensor. 0: train live, 1: train fake samples from CrossMatch sensor, 2: test live, 3: test fake samples from Biometrika sensor.

Figure 7 and Figure 8 show the distribution of fingerprint samples before adaptation. From the figures, we can see a distribution shift between the train samples and test samples in the case of the cross-sensor scenario, which causes degradation in accuracy.

#### 3.3.3. Experiment 3: Cross-Sensor Performance 

In this experiment, we evaluated the proposed method to detect and prevent PAs in cross-sensor and cross-material scenarios. The proposed method is divided into two steps. The first one is to synthetically generate additional images by translating the images from the source domain (one sensor) to the target domain (multiple sensors) using a CycleGAN model. The output of this step is combined with the source domain complete dataset to form a new dataset. The second step is to train the architecture of the CNN backbone and transformer on the new dataset. Later we tested the trained model on images from the testing part of LivDet 2015 dataset.

We trained the CycleGAN to synthetically generate additional images using a randomly selected image from one sensor (source domain) and images from other sensors (target domain). For example, we randomly selected 40 real and 40 fake images from Green Bit and considered it a source domain. Then, we randomly selected 20 real images from each of the other sensors and considered it the target domain. We combined the dataset generated from CycleGAN with the original dataset and retrained the proposed network for sufficient 20 iterations. 

The reported results in Table 5 show the improvement in classification accuracy in cross-sensor setting. For example, when we retrained the proposed network on original images from source domain (Crossmatch sensor) and the generated dataset from CycleGAN, the classification accuracy increased by 9.28%, 6.2%, and 10.38% for testing the network using Green Bit, Biometrika, and Digital Persona, respectively. 

**Figure 9:**
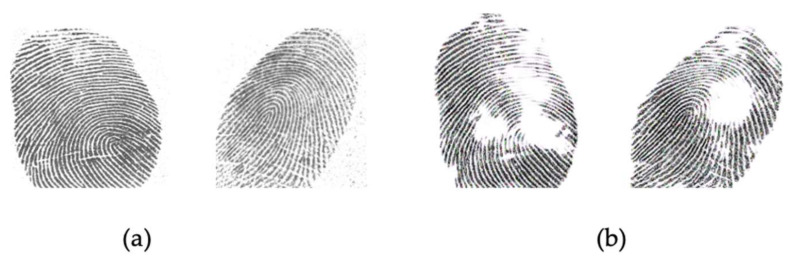
Image conversion from GreenBit domain (**a**) to the joint domains (**b**) of other sensors.

**Figure 10:**
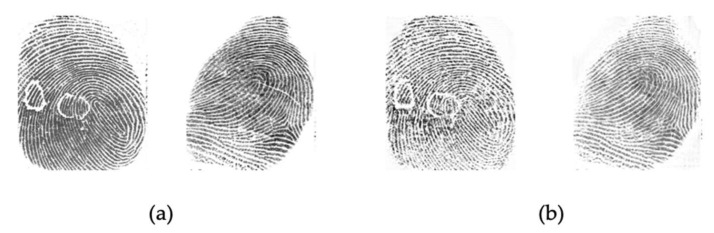
Image conversion from Crossmatch domain (**a**) to the joint domains (**b**) of other sensors.

Figure 9 and Figure 10 show samples of the conversion from one domain to the joint domains of other sensors. We can see that the generated image style is imitating the style of the target domain. The generated images are considered as new images of the Livdet2015 dataset and used in this experiment.

**Figure 11:**
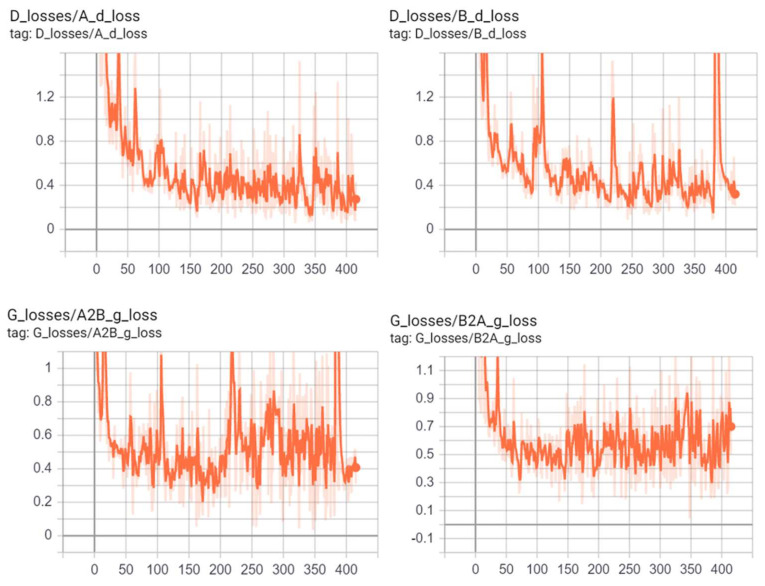
Example of discriminators and generators losses of CycleGAN during learning.

Figure 11 shows the evolution of the training loss of the generator and discriminator of CycleGAN on the Livdet2015 dataset over epochs. 

## 4. Discussion

Going deeper, we repeated the experiments using different configurations of the core architecture of the CycleGAN. We replaced the regular GANs architecture of the generator and discriminator with least squares generative adversarial networks (LSGANs) [42]. The LSGAN is a GAN model that adopts the least-squares loss function instead of the sigmoid cross-entropy loss for the discriminator, which provides a smooth and nonsaturating gradient in the discriminator. The LSGAN objective function is described below:

(13) $$\underset{D}{\min}V_{LSGAN}\left(D\right)\;=\;\frac{1}{2}E_{x∼P_{\mathrm{data}}\left(x\right)}\left[{\left(D\left(x\right)-b\right)}^{2}\right]+\frac{1}{2}E_{z∼P_{z}\left(z\right)}[(D{\left(G\left(z\right))-a\right)}^{2}]$$

(14) $$\underset{G}{\min}V_{LSGAN}\left(G\right)\;=\;\frac{1}{2}E_{z∼P_{z}\left(z\right)}[(D{\left(G\left(z\right))-c\right)}^{2}]$$

where *a* and *b* are the labels for fake data and real data, and c the value that *G* wants *D* to believe for fake data. The least-squares loss function is used to penalize the fake samples by pulling them toward the decision boundary close to the real data distribution. Based on this property, LSGAN can generate high-quality images and perform more stably during the learning process. Despite that, from the experimental result in Table 6 we can see that using LSGAN instead of regular GAN did not significantly change the results.

## 5. Conclusions

In this work, we proposed a fingerprint presentation attack method to increase the detection accuracy and the generalization ability against unknown sensors and materials. The proposed method is based on pretrained CNN and vision transformer combined with CycleGAN. We performed several experiments with different scenarios to validate and evaluate the proposed methodologies and all the experiments conducted on the LivDet2015 dataset. The experimental results showed the importance of dataset augmentation in increasing the classification accuracy. For future developments, we propose to investigate other GAN-based models to increase the detection accuracy.

## Figures and Tables

**Table 1:** Summary of LivDet2015 datasets used in the experiments.

Sensor	Model	Resolution (dpi)	Image Size (px)	# of Training (Live/Spoof)	# of Testing (Live/Spoof)
Green Bit	DactyScan26	500	500 × 500	1000/1000	1000/1500
Biometrika	HiScan-PRO	1000	1000 × 1000	1000/1000	1000/1500
Digital Persona	U.are.U 5160	500	252 × 324	1000/1000	1000/1500
Crossmatch	L Scan Guardian	500	640 × 480	1500/1500	1500/1448

**Table 2:** Spoof materials used in LivDet2015 dataset.

Sensor	Spoof Material Used in Training	Spoof Material Used in Testing
Green Bit	Ecoflex, gelatin, latex, and wood glue	Ecoflex, gelatin, latex, wood glue, liquid Ecoflex, and RTV
Biometrika		
Digital Persona		
Crossmatch	Play-Doh, Body Double, and Ecoflex	Play-Doh, Body Double, Ecoflex, OOMOO, and Gelatin

**Table 3:** Comparison between the results of the proposed network and the state-of-the-art approaches on LivDet 2015 dataset in terms of classification accuracy (acc %).

Algorithm	Green Bit	Biometrika	Digital Persona	Crossmatch	Average
Nogueira (first place winner) [38]	95.40	94.36	93.72	98.10	95.40
Unina (second place winner) [38]	95.80	95.20	85.44	96.00	93.11
Zhang et al. [41]	97.81	97.02	95.42	97.01	96.82
M. Jomaa et al. [15]	94.68	95.12	91.96	97.29	94.87
Proposed network [no-augmentation, p=7]	96.72	95.44	94.76	98.30	96.31
**Proposed network** **[simple augmentation**, p=7] *	**98.44**	**97.68**	**96.36**	98.33	**97.70**
Proposed network [cutmix augmentation, p=7]	97.48	97.64	94.40	**99.01**	97.13
Proposed network [simple augmentation, p = 4]	97.00	95.84	91.11	98.50	95.61

* The best performance results are highlighted in bold.

**Table 4:** The generalization performance among cross-sensors on LivDet 2015 in terms of classification accuracy (acc%) and average classification error (ACE).

	Sensor in Testing	Green Bit	Biometrika	Digital Persona	Crossmatch	Average
Sensor in Training						
Green Bit	Acc	97.56	83.68	66.60	63.97	77.95
	ACE	2.23	20.20	41.75	35.47	24.91
Biometrika	Acc	80.12	94.80	87.28	57.86	80.02
	ACE	16.76	4.70	12.33	42.81	19.15
Digital Persona	Acc	52.40	70.36	91.00	60.31	68.52
	ACE	39.78	25.13	8.00	40.36	28.32
Crossmatch	Acc	70.76	70.04	50.32	97.79	72.23
	ACE	26.90	29.08	44.86	2.17	25.75

**Table 5:** The proposed network’s performance on LivDet 2015 in terms of classification accuracy (acc%) and average classification error (ACE).

	Sensor in Testing	Green Bit	Biometrika	Digital Persona	Crossmatch	Average
Sensor in Training						
Green Bit	Acc		91.20	81.20	76.96	83.12
	ACE		10.20	23.21	23.06	18.82
Biometrika	Acc	89.52		86.72	69.77	82.00
	ACE	9.81		15.30	30.62	18.58
Digital Persona	Acc	85.36	84.96		69.02	79.78
	ACE	13.05	14.28		31.36	19.56
Crossmatch	Acc	80.04	76.24	60.70		72.33
	ACE	17.43	22.61	35.51		25.18

**Table 6:** Comparison between the results of the proposed network without using GAN, using GAN and using LSGAN on LivDet 2015 dataset in terms of classification accuracy (acc %).

	Sensor in Testing	Green Bit	Biometrika	Digital Persona	Crossmatch	Average
Sensor in Training						
Green Bit	No-GAN		83.68	66.60	63.97	71.42
	GAN		91.20	81.20	76.96	83.12
	LSGAN		90.52	80.16	72.62	81.10
Biometrika	No-GAN	80.12		87.28	57.86	75.09
	GAN	89.52		86.72	69.77	82.00
	LSGAN	92.76		88.08	66.55	82.46
Digital Persona	No-GAN	52.40	70.36		60.31	61.02
	GAN	85.36	84.96		69.02	79.78
	LSGAN	85.88	83.20		69.17	79.42
Crossmatch	No-GAN	70.76	70.04	50.32		63.71
	GAN	80.04	76.24	60.70		72.33
	LSGAN	80.10	75.78	61.00		72.29
